# Evasion of Antimicrobial Activity in *Acinetobacter baumannii* by Target Site Modifications: An Effective Resistance Mechanism

**DOI:** 10.3390/ijms23126582

**Published:** 2022-06-13

**Authors:** Arturo Martínez-Trejo, Juan Manuel Ruiz-Ruiz, Luis Uriel Gonzalez-Avila, Andrés Saldaña-Padilla, Cecilia Hernández-Cortez, Miguel Angel Loyola-Cruz, Juan Manuel Bello-López, Graciela Castro-Escarpulli

**Affiliations:** 1 Laboratorio de Investigación Clínica y Ambiental, Departamento de Microbiología, Escuela Nacional de Ciencias Biológicas, Instituto Politécnico Nacional, Prolongación de Carpio y Plan de Ayala s/n, Col. Casco de Santo Tomas, Miguel Hidalgo, Ciudad de Mexico 11340, Mexico; arturo.martinezt@outlook.com (A.M.-T.); u_gza@hotmail.com (L.U.G.-A.); andres950123@hotmail.com (A.S.-P.); miguelqbp@gmail.com (M.A.L.-C.); 2 Laboratorio Clínico, Unidad Médica de Alta Especialidad, Hospital de Pediatría Dr. Silvestre Frenk Freund, Centro Médico Nacional Siglo XXI, Instituto Mexicano del Seguro Social, Av. Cuauhtémoc 330, Col. Doctores, Ciudad de Mexico 06720, Mexico; ruizjm06@hotmail.com; 3 Laboratorio de Bioquímica Microbiana, Departamento de Microbiología, Escuela Nacional de Ciencias Biológicas, Instituto Politécnico Nacional, Prolongación de Carpio y Plan de Ayala s/n, Col. Casco de Santo Tomas, Miguel Hidalgo, Ciudad de Mexico 11340, Mexico; cecihercor@hotmail.com; 4 División de Investigación, Hospital Juárez de Mexico, Av. Instituto Politécnico Nacional 5160, Magdalena de 11 las Salinas, Gustavo A. Madero, Ciudad de Mexico 07760, Mexico; juanmanuelbello81@hotmail.com

**Keywords:** *Acinetobacter baumannii*, antimicrobial resistance, mechanisms of antimicrobial resistance, target site modification

## Abstract

*Acinetobacter baumannii* is a Gram-negative bacillus that causes multiple infections that can become severe, mainly in hospitalized patients. Its high ability to persist on abiotic surfaces and to resist stressors, together with its high genomic plasticity, make it a remarkable pathogen. Currently, the isolation of strains with high antimicrobial resistance profiles has gained relevance, which complicates patient treatment and prognosis. This resistance capacity is generated by various mechanisms, including the modification of the target site where antimicrobial action is directed. This mechanism is mainly generated by genetic mutations and contributes to resistance against a wide variety of antimicrobials, such as β-lactams, macrolides, fluoroquinolones, aminoglycosides, among others, including polymyxin resistance, which includes colistin, a rescue antimicrobial used in the treatment of multidrug-resistant strains of *A. baumannii* and other Gram-negative bacteria. Therefore, the aim of this review is to provide a detailed and up-to-date description of antimicrobial resistance mediated by the target site modification in *A. baumannii*, as well as to detail the therapeutic options available to fight infections caused by this bacterium.

## 1. Introduction

Antimicrobial resistance is a worrying and growing problem [1,2]. In the USA, it is estimated that antibiotic-resistant microorganisms cause more than one million infections each year, which is linked to at least 23,000 deaths. It is estimated that by the year 2050, the number of deaths will increase tenfold [3]. In recent years, the World Health Organization (WHO) has recommended global action to reduce the presence of antibiotic resistant isolates in all countries; carbapenem-resistant *Acinetobacter baumannii* is a microorganism that is a priority for research and development of new treatments [4]. Currently, there are more than 50 designated species of *Acinetobacter*, of which the vast majority are considered non-pathogenic. The most clinically important species of the genus *Acinetobacter* are within the *Acinetobacter calcoaceticus-baumannii* (Acb) complex [5]. The Acinetobacter complex is composed of five pathogenic species (*A. baumannii, Acinetobacter nosocomialis, Acinetobacter pittii, Acinetobacter seifertii*, and *Acinetobacter dijkshoorniae*) and a non-pathogenic member (*Acinetobacter calcoaceticus*) [5,6]. *A. baumanni* is the most relevant and most studied species; it is considered an opportunistic human pathogen. A total of 2% of HCAIs (healthcare-associated infections) are caused by this microorganism [6]. It is estimated that 45% of isolates worldwide are multidrug-resistant (MDR), reaching percentages of up to 70% in Latin America and the Middle East [7]. This bacterium can cause several HCAIs [8]. One of the most frequent with the highest mortality rates is ventilator-associated pneumonia and bacteremia; these infections are directly related to patients with comorbidities or in critical condition. Infections caused by this pathogenic carrier have been observed in patients with prolonged periods of hospitalization [9,10]. As mentioned above, *A. baumannii* has been detected in sputum cultures and tracheal aspirates from COVID-19 mechanically ventilated patients. This opportunistic pathogen is responsible for approximately 47% of the cases of monomicrobial ventilator-associated pneumonia (VAP) infections in the ICU (intensive care unit); however, other bacterial species are also related to this infection, such as *Pseudomonas aeruginosa*, *Staphylococcus aureus*, and some members of the *Enterobacterales* group; even co-infections between the aforementioned microorganisms have been reported. Patients with impaired COVID-19 present the main risk factors for developing VAP caused by *A. baumannii*, which are hypertension, chronic obstructive pulmonary disease, chronic renal failure, and prolonged stay in the ICU [11,12,13,14,15,16,17]. Many reports have shown that *A. baumannii* rapidly develops antimicrobial resistance [18]. Several mechanisms of resistance have been reported in this microorganism, such as the enzyme-mediated degradation of antibiotics, modifications of target sites, efflux pumps, and changes in membrane permeability [19]. In this review, we focus on the different mechanisms of resistance to the main groups of antibiotics and antimicrobial therapy in infections caused by *A. baumannii*.

## 2. Current Status of Antimicrobial Resistance in *A. baumannii*

Currently in the clinic, the isolation of *A. baumannii* is of relevance and has become a serious problem, mainly due to the ability of this bacterium to acquire and regulate various resistance determinants, which has made it one of the most successful pathogens in colonization and infection [20]. This success is also due to its ability to resist the action of different antimicrobials, as described below.

In the literature, there are several reports on the resistance of this bacterium to different families of antimicrobials, such as β-lactams, which mainly occur by the presence of β-lactamases. It has been described that *A. baumannii* presents the four types of these enzymes proposed by Ambler (classes A, B, C, and D). Strains that present class A enzymes show resistance to all penicillins and cephalosporins, except cephamycins. Within the above, reports stand out where it is mentioned that this bacterium presents KPC enzymes (KPC-2, KPC-3, and KPC-5) [21,22,23]. For strains that present class B enzymes, known as MBLs, they have resistance to all β-lactams, including carbapenem [24]; this highlights the presence of NDM-1 enzymes reported in various parts of the world, such as Iran, China, Tunisia, Saudi Arabia, and Lebanon [23].

Class C enzymes, known as acinetobacter-derived cephalosporinases (ADCs), which are intrinsic to all *A. baumannii*, confer resistance to cefoxitin, cefotenan, cephalosporins, and penicillin. In the case of class D enzymes or oxacilinases (OXAs), it is known that, in *A. baumannii*, they confer resistance to carbapenem; these are mainly found encoded in plasmids [19,24,25]. Currently, it is known that, in *A. baumannii*, apart from the resistance to β-lactams presented by these enzymes, there may be non-enzymatic mechanisms that confer resistance against this type of antimicrobials, such as changes in the porins of their membranes, such as CarO, which is associated with resistance to imipenem and meropenem [26]. Not only are porins related to this resistance, but also efflux pumps that generate resistance against several β-lactams, aminoglycosides, erythromycin, chloramphenicol, fluoroquilones, tetracyclines, and trimethoprim [27].

Regarding antimicrobials that do not belong to the β-lactam family, it is known that this bacterium can present resistance to tetracyclines and glycylcyclines for the first case involving efflux pumps where RND pumps participate [28]. There are reports where there are strains with resistance to tigecycline, related to efflux pumps [29].

*A. baumannii* also presents resistance to fluroquinolones. The mutations in the *gyrA* gene are related to resistance to fluoroquinolones; however, there are other mechanisms of resistance to these antibiotics, such as efflux pumps, which do not have a broad spectrum on all particular antibiotics of the fluoroquinolone group; this spectrum is reduced only to ciprofloxacin and norfloxacin [30].

There is evidence of resistance to aminoglycosides in this bacterium, mainly given by the participation of enzymes with the activity of acetyltransferases, adenyltransferases, and phosphotransferases, which causes resistance to amikacin. Likewise, there are changes in ribosomal target sites where the action of these antimicrobials is directed, which can provide resistance to gentamicin, trobamycin, and amikacin [31], as well as the involvement of efflux pumps, where the action of gentamicin is affected [32].

Regarding macrolides, there are reports of resistance to azithromycin, erythromycin, and chloramphenicol, although some strains of *A. baumannii* show variable resistance to azithromycin [33].

In terms of polymyxin resistance, colistin resistance stands out, as it is a rescue antimicrobial, which, in recent years, has considerably increased [34].

### Intrinsic Resistance in A. baumannii

Intrinsic resistance is that which is innate in bacteria, which is not acquired, but occurs naturally due to the characteristics of the bacteria themselves. This type of resistance is reflected in all or most wild-type strains [19,35].

Knowing this type of resistance is relevant in the clinic to avoid ineffective treatments as well as performing susceptibility testing that will be unnecessary [35,36].

A clear example of intrinsic resistance in *A. baumannii* is towards β-lactams due to different causes, for example, in the chromosome of this bacterium there are bla*_OXA_*, bla*_ADC_*, and bla*_AmpC_* genes encoding for β-lactamases. In addition, naturally, there are alterations in PBPs, there is also the presence of efflux pumps naturally found in the bacterium, such as the RND family, and finally, it is known that there are changes in the membrane permeability of this bacterium that lead to resistance [19,25,37,38,39,40].

One study showed that intrinsic resistance in *A. baumannii* can be affected when there are mutations leading to a deficit in the production of capsular polysaccharides [41].

The US Clinical and Laboratory Standard Institute (CLSI) reports that this bacterium is intrinsically resistant to ampicillin, amoxicillin, amoxicillin with clavulanate, aztreonam, ertapenem, trimethoprim, chloramphenicol, and fosfomycin (Table 1) [36].

## 3. Mechanisms of Antimicrobial Resistance

There are several mechanisms used by bacteria to evade the effect of antimicrobials. Among these are the inactivation of these compounds by their modification and/or degradation, limiting the antimicrobial to reach its target site by efflux/expulsion or preventing its entry into cells by decreasing the permeability of membranes or sequestering their molecules and changing or altering the target sites to which the action of antimicrobials is directed [35,45,46].

### 3.1. Antimicrobial Modification

The modification and/or degradation of antibiotics is one of the most common strategies used by microorganisms to evade the action of these drugs. The participation of various enzymes can cause the hydrolysis of the antimicrobial; transfer functional groups such as acyls, glycosyls, nucleotidyls, phosphoryls, or thiols; or perform oxidation-reduction processes leaving the antimicrobial molecules unusable [46].

An example of these enzymes is the β-lactamases, which are the most studied enzymes and whose relevance is explained, since the antimicrobials of the β-lactam family are an extensive group of compounds widely used in the clinic. Several of these were the product of the development and modification of penicillin, so that, as these new antimicrobials were generated, new enzymes that had the ability to degrade them were discovered as, for example, the extended spectrum β-lactamases or ESBLs, which can degrade several types of β-lactams (for example, the KPC enzymes); there are also metallo-β-lactamases or MBLs, such as the New Delhi variant or NDM, and OXA lactamases where carbapenemases are found [46,47,48,49,50].

### 3.2. Antimicrobial Efflux and Decreased Permeability

The efflux or expulsion of antibiotics is another common mechanism that bacteria use to avoid the action of antimicrobials. For this mechanism, bacteria make use of carrier proteins that carry the antibiotic out of the cells, known as carriers. However, this mechanism also involves a decrease in the permeability of the bacterial membrane [49].

In this classification, we find the efflux pumps that have been described in Gram-positive and Gram-negative bacteria, which are classified into five families: ABC, RND or resistance–nodulation–division, MFS, MATE (multidrug and toxin extrusion), and SMR (small multidrug resistance). Only the ACB type requires the participation of ATP as energy to transport antibiotics; the others use an ion gradient, and this type of pump has been well described in strains of *E. coli*, *S. aureus*, and *L. lactis strains*. This mechanism reduces the concentration of antibiotics within the bacterial cytoplasm and the minimum inhibitory concentration (MIC) of antibiotics needed for it to take effect cannot be reached [46,50].

To prevent the antimicrobial from entering the cells, bacteria can generate the decrease in permeability in their membranes where the chemical nature that composes the bacterial membranes intervenes, such as the nature of lipopolysaccharides. However, the participation of porins, which are selective molecules to the passage of molecules from the outside to the inside of the cells, which limits the entry of certain antimicrobials, stands out; examples of this resistant mechanism are the CarO and the OmpA porins in *A. baumannii*. The OmpA porin has been associated with decreased minimum inhibitory concentrations of chloramphenicol, aztreonam and nalidixic acid, and the CarO porin with resistance to carbapenems [19,46,51,52].

### 3.3. Antimicrobial Sequestration

The sequestration of antibiotics is a mechanism that prevents them from reaching their site of action. For this, the participation of proteins that bind to these compounds is necessary, for example, the TlmA, BlmA, and ZbmA proteins that generate resistance to bleomycin. This is well known in *S. hindustanus*, *S. verticillus*, and *Streptomyces flavoviridis*, respectively. In this case, each bleomicyn family needs one or more genes related to the transporters ABC and grouped in clusters, which are products used to remove the antibiotics bound to binding proteins [50,51,52].

### 3.4. Modification of the Target Site

The alteration of target sites is a mechanism of bacterial resistance to antimicrobials. In most bacterial groups of clinical origin, there are several mechanisms of resistance, including *A. baumannii* [53]. Target site changes are generally due to mutational processes in the bacterial genome; these mutations happen spontaneously in the genes coding for the target sites and often occur due to selective pressure phenomena in the presence of the antimicrobial [19,54]. The most frequent changes are mutations in the RNA polymerase and DNA gyrase genes, causing the bacterium to become resistant to rifampicin and quinolones, respectively [55].

In *A. baumannii*, little has been described on the mechanisms of resistance due to the change in target site, however, the best described in this genus is the decrease in the affinity of penicillin-binding protein type 2 (PBP 2) towards imipenem. Nevertheless, other factors, such as membrane permeability or the presence of efflux pumps, could add to the resistance [56].

The simplest way in which the mechanisms of resistance mediated by a change in the target site can be classified is to divide them into four main groups. In the case of *A. baumannii*, and other bacteria members of the ESKAPE group, the first is the mechanism of antimicrobial inactivation mediated by modifying enzymes that are responsible for irreversibly destroying the antimicrobial target site, for example, the hydrolytic cleavage of the β-lactam ring. Additionally, the structures of the key sites for antimicrobial anchorage can be covalently modified, which hinders the antimicrobial/target site interaction. Included in this group is 16S RNA methylation [53].

The second group refers to the modification of the target site structure that decreases the affinity of the structure for the antimicrobial preventing binding at the cell surface; the typical mechanism is LPS modification, PBP 2 protein modification, and the peptidoglycan modification [57,58,59].

The third group includes the phenomenon by which the bacteria prevent the antimicrobial from accumulating inside the cell; this is due to the mutation or total loss of the channels of the outer membrane, the CarO type in *A. baumannii*, in addition to the efflux systems of RND, MFS, ABC, among others. The fourth and last group refers to the persistence process and biofilm formation. In the first one, the bacteria become tolerant to the presence of the antimicrobial when phenotypic changes, such as a quiescent metabolism, occur in small subpopulations [53]. The second one is affected by the penetration and activity of the antibiotic, making the bacteria inside the biofilm matrix more resistant compared to those in a sessile state [60,61,62].

## 4. Antimicrobials Whose Effect Is Evaded by Target Site Modification in *A. baumannii*

*Acinetobacter baumannii* uses three mechanisms by which it shows antimicrobial resistance. The first one prevents the antimicrobial to reach the target site where it directs its action and occurs through the presence of efflux pumps or reducing the permeability of the membrane. The second mechanism occurs through the inactivation of antimicrobials, where these can be modified in some part of their structure or hydrolyzed. In this mechanism, the participation of various enzymes is involved, mainly carbapenemases. The third mechanism by which *A. baumannii* presents resistance is through the modification of the target site to which the antimicrobial is directed, which is given by genetic mutations in specific sites or by post-transcription modifications in various proteins [59,62].

**Figure 1:**
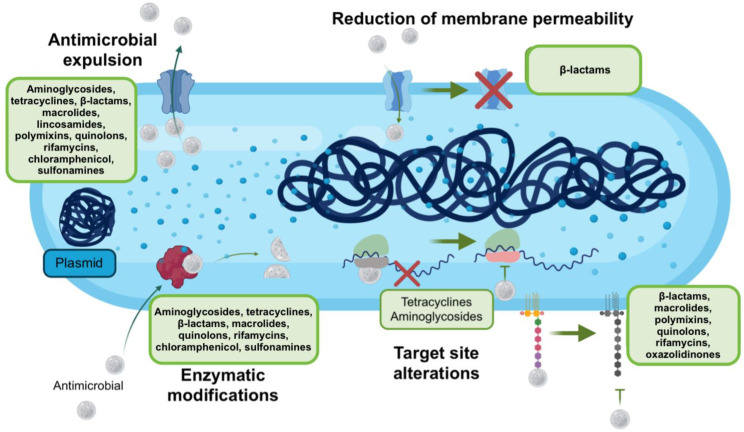
Mechanisms used by *A. baumannii* to evade the action of antimicrobials. *A. baumannii* use various mechanisms to prevent antimicrobials from causing damage, among which are the expulsion of these compounds, the decrease in permeability so that they cannot penetrate, as well as the modification of their target sites or the degradation of these compounds by enzymatic action. Taken and modified from [61]. Created with BioRender.com (accessed on 3 June 2022).

This last mechanism is responsible for generating a wide resistance to various antimicrobials, such as fluoroquinolones, tetracyclines, macrolides, oxazolidinones, aminoglycosides, rifamycin, polymyxins, and β-lactams [59]. For this reason, studying this mechanism is of interest for combating resistant strains of *A. baumannii* (Figure 1).

### 4.1. Polymyxin Resistance

The increasing emergence of MDR strains of *A. baumannii* makes this bacterium a global health problem. Because of the difficulty in treating *A. baumannii* infections with high-antimicrobial-resistance profiles, there are alternative antimicrobials, such as polymyxins. Polymyxins are polycationic peptide antibiotics that are used in the treatment of Gram-negative bacterial infections. This group of antibiotics consists of five different types of compounds (polymyxins A to E) for which the spectrum is narrow. In the treatment of *A. baumannii* infections, the use of polymyxins B and E (colistin) is well-known. Because of the increased use of these salvage or last alternative antimicrobials, the phenomena of resistance to these have increased, making it difficult to treat and improve patients [63,64,65,66].

Polymyxin B has adequate activity in vitro, however, in vivo treatments have been ineffective, especially in systemic infections, so its therapeutic use is avoided, in addition to the probable cytotoxic damage, so polymyxin E or colistin is preferred [19].

In recent years, strategies have been implemented for the treatment of infections caused by *A. baumannii* with high-antimicrobial-resistance profiles. Such strategies include the combination of treatment with tigecycline, carbapenems, or rifampicin, which, at some point, was efficient; however, the emergence of the phenomena of heteroresistance and resistance to colistin, per se, has been reported with a significant increase in recent years, becoming a treatment failure [62].

#### Colistin Resistance in *A. baumannii*

Resistance to colistin in *A. baumannii* has been described in several in vitro and in vivo studies, in which it has been determined that there are mechanisms by genetic changes that induce resistance to colistin by the modification of lipopolysaccharides (LPSs), mainly due to single nucleotide polymorphisms (SNPs) in pmrAB protein systems [67]. Another likely mechanism of colistin resistance is the loss of LPSs by SNPs in the lipid biosynthesis genes *lpxA*, *lpxC*, and *lpxD*. Due to the latter, the negative charges interacting with colistin, important in the entry mechanism of this antimicrobial, are lost [57]. Additionally, insertion sequence elements (IS), such as ISAba1 and ISAba11, have been associated with the development of colistin resistance through the spontaneous mutation of *lpx* genes [62].

The main mechanism of colistin resistance Is mediated by *mcr* genes. In *A. baumannii*, the presence of *mcr* genes has not been reported; however, up to this moment, colistin-resistant isolates have increased considerably. In *A. baumannii*, the mechanism mediated by the mutation of the *pmrA* and/or *prmB* protein genes is supposed to be the most common, which, together with the constitutive expression of *prmA*, causes the positive regulation of the *pmrCAB* operon and the addition of phosphoethanolamine to the phosphate of LPSs. Interestingly, mutations in *pmr* genes are usually reversible; this by compensatory mutation in the *pmr* locus that decreases the activity of the *pmrCAB* operon. Despite this, some strains can maintain the resistant phenotype, which would explain the presence of other mechanisms or that mutations occur in other genes different from the *pmr* complex; however, the alternative mechanism has not been determined [57,62].

### 4.2. Resistance to β-Lactams

The production of β-lactamases is the main mechanism of resistance in *A. baumannii*. The enzymes mostly reported are the cephalosporinase type encoded on the type 1 chromosome, which conifers resistance to first- and second-generation cephalosporins [55].

Some reports have described the production of extended spectrum β-lactamases (ESBLs) in MDR strains of *A. baumannii*, but this assertion has not been confirmed. Other less frequently reported in this genus are the ARI-1 and OXA types encoded in plasmids. In contrast, the production of beta-lactamases is favored when the external membrane is not very permeable or by the decrease in the transporter proteins in the external membrane. Resistance to beta-lactams may also be due to the decreased expression of porins, whereby the antimicrobial cannot enter the interior of the bacterium [68].

As it is known, the production of β-lactamases and the decrease in the expression and changes in the porins’ structure are key factors for β-lactam resistance [59,68]. However, they are not the only mechanisms that confer resistance to β-lactams in *A. baumannii*. Structural alterations are also found in penicillin-binding proteins (PBPs), which are the target of some β-lactams [59,66]; if PBPs are changed, the effect of β-lactams is reduced. PBPs are crucial for cell wall biosynthesis during cell proliferation [69,70]. 

These proteins are classified into two groups based on their molecular mass (high-molecular-mass PBPs (HMM) and low-molecular-mass PBPs (LMM)), which have been detected in carbapenem-resistant *A. baumannii* isolates. PBPs are responsible for catalyzing transglycosylation and cross-linking by peptidoglycan transpeptidation, generating the cell wall polymer. It has been reported that the modification of PBPs could be considered as one of the mechanisms of carbapenem resistance [52]. The modification of PBPs and related effects on β-lactam resistance was previously reported in *Escherichia coli* K-12, *Pseudomonas aeruginosa*, and *Streptococcus pneumoniae*. Modifications of PBPs have been shown to play an important role in β-lactam resistance in Gram-negative bacteria, including *A. baumannii* [71].

### 4.3. Rifampicin Resistance

Resistance to rifampicin (also known as rifabicin) in *A. baumannii* infections has been linked to mutations in the *rpoB* gene, which encodes the β-subunit of rifamycin-sensitive RNA polymerase and prevents RNA elongation just after the first nucleotides are added. Beyond rifampicin, RpoB is associated with resistance to all rifamycins (rifabutin, rifaximin, and rifapentine) [59].

Rifampicin binds to the active site of bacterial RNA polymerase, inhibiting the transcription process. The mechanism of evasion of this drug is amino acid substitution in the β-subunit of this target protein [72,73].

The binding site between rifampicin (also known as rifamycin) and the β-subunit of RNA polymerase is highly conserved among bacteria. The binding of the molecule to RNA polymerase involves 12 amino acid residues. Rifampicin binds to the active site of bacterial RNA polymerase, inhibiting the transcription process. The mutagenesis of each of these residues, except one, generates a resistant phenotype. In *A. baumannii* infections, it has been linked to mutations in the *rpoB* gene, which encodes the β-subunit of rifamycin-sensitive RNA polymerase and prevents RNA elongation just after the first nucleotides are added. Beyond rifampicin, RpoB is associated with resistance to all rifampicins (rifabutin, rifaximin, and rifapentine) [55,68,69]. Spontaneous rifampicin resistance is primarily associated with single-point mutations resulting in amino acid substitutions, and it is less frequently associated with some insertions or deletions. Ninety-five percent of these mutations map to four regions in the N-terminal half of the polypeptide subunit involved in rifampicin binding [74].

### 4.4. Resistance to Fluoroquinolones

Quinolones are broad-spectrum bactericides characterized by a bicyclic core formation resembling 4-quinolone [59]. Fluoroquinolones act by inhibiting DNA gyrase and topoisomerase IV, two enzymes involved in DNA synthesis. DNA gyrase introduces negative superhelical turns into the DNA double helix before the replication fork. It comprises two GyrA and two GyrB subunits, encoded by the *gyrA* and *gyrB* genes, respectively. Topoisomerase IV is responsible for the decanting of the daughter chromosomes produced at the end of a round of replication. It comprises two ParC subunits and two ParE subunits encoded by the *parC* and *parE* genes, respectively [54].

Quinolones and fluoroquinolones act by binding to the enzyme gyrase (encoded by the *gyrA* and *gyrB* genes) and topoisomerase IV (encoded by the *parA* and *parC* genes). At first, when these bind to the DNA, a complex made up of quinolone–enzyme–DNA is formed; this is similar to the case of topoisomerases [69], and this could lead to creating conformational changes that result in the inhibition of normal enzyme activity. As a result, the bacteria cannot perform the DNA replication process and die. The ternary complexes of drugs, enzymes, and DNA block the progress of the replication fork. The action of fluoroquinolones results from the conversion of the topoisomerase–quinolone–DNA complex into an irreversible form and the generation of double-strand breaks in DNA by the denaturation of topoisomerase [54].

Resistance to fluoroquinolones is mainly mediated by spontaneous mutations of genes in the quinolone resistance determinant region (QRDR), namely, DNA gyrase and topoisomerase IV. Alterations in the drug target due to modifications in DNA gyrase subunit A (*gyrA*) or topoisomerase IV subunit C (*parC*) genes have been associated with high levels of resistance to fluoroquinolones. The mechanism of fluoroquinolone resistance in *A. baumannii* consists of substitutions in the QRDR of DNA gyrase and DNA topoisomerase IV, which interfere with the binding of fluoroquinolones to their target proteins [72]. QRDRs mainly concern altered target sites in gyrase (Ser83Leu, Gly81Asp, and Ser81Leu mutations that prevent quinolones from binding to their α-subunit) and topoisomerase IV (Ser80Leu, Glu84Lys, and Gly78Cys mutations, and Ser84Leu in their C subunit) [55]. The most common amino acid codon mutations leading to fluoroquinolone resistance in *A. baumannii* occur at Ser 83 and Gly 81 within gyrA, and Ser 80 and Glu 84 within parC [71,72,73].

Mutations in the target sites of quinolones and fluoroquinolones have also been widely reported in *A. baumannii*; mutations resulting in a Ser-86-Leu substitution in GyrA and a Ser-80-Leu substitution in ParC increase MIC of ciprofloxacin in clinical isolates [69]. For example, clinically significant resistance to fluoroquinolones can be achieved with a single mutation in *gyrA*, as observed in the study conducted by Esterly et al. in 2011, in which MICs of ciprofloxacin increased from 0.38 mg/L to 12 mg/L. However, double amino acid substitutions in the *gyrA* and *parC* genes are required for a higher level of resistance (MIC of ciprofloxacin > 32 mg/L). Similarly, in the study conducted by Lin et al., in 2010, 85% of *A. baumannii* isolates (45/53) were resistant to fluoroquinolones, and all of these contained a Ser83Leu mutation in GyrA, except for one ciprofloxacin-resistant isolate with Ser83 in GyrA that was susceptible to levofloxacin. Only 55% (24/44) of the previously described *A. baumannii* isolates with a Ser83Leu mutation in GyrA were also resistant to levofloxacin, and 43% (19/44) were intermediate to levofloxacin [74,75,76,77,78,79].

### 4.5. Macrolides Resistance

Macrolide antibiotics are polyketides composed of a 14-, 15-, or 16-membered macrocyclic lactone ring (14-, 15-, and 16-membered) to which various sugars and/or side chains have been attached by the producing organism or as modifications during semi- synthesis in the laboratory [79]. The macrolide, lincosamide, and streptogramin B group of antibiotics block protein synthesis in bacteria by binding to the 50S ribosomal subunit. Resistance to these antibiotics is known as MLSB-type resistance and occurs in a wide range of Gram-positive and Gram-negative bacteria. It results from a post-transcriptional modification of the 23S rRNA component of the 50S ribosomal subunit involving methylation or dimethylation of key adenine bases in the peptidyl transferase functional domain. Methylation is catalyzed by adenine-specific N-methyltransferases specified by the *erm* (erythromycin ribosome methylation) class of genes, present in a wide range of organisms, and frequently encoded by plasmids [54].

Erm methyltransferases add one or two methyl groups to the exocyclic N-6 amino group of A2058, disrupting the key hydrogen bond between A2058 and the desosamine sugar at C5. Ribosomal methylation by methyltransferases encoded by *erm* genes remains the most widespread macrolide resistance in pathogenic bacteria, and certain *erm* genes are predominantly found in some species [79]. Residue A2058 lies within a conserved region of the 23S ribosomal RNA domain V, which plays a key role in the binding of MLSB antibiotics. As a consequence of methylation, the binding of erythromycin to its target is altered. Overlapping binding sites of macrolides, lincosamides, and streptogramin B on 23S rRNA explain cross-resistance to all 3 drug classes. A wide range of microorganisms that are targets of macrolides and lincosamides, including Gram-positive species, spirochaetes, and anaerobes, express Erm methylases. Nearly 40 *erm* genes have been reported to date. In pathogenic bacteria, these determinants are mainly supported by plasmids and transposons that are self-transferable. A nomenclature system has been designed to avoid further complexity in naming. *Erm* genes with a deduced amino acid sequence identity of <80% have different letter designations. This new nomenclature distinguishes 21 classes of *erm* genes and as many corresponding Erm proteins. Four main classes are detected in pathogenic microorganisms: *erm(A)*, *erm(B)*, *erm(C)*, and *erm(F)*. The fact that each class is relatively specific, but not strictly limited to a bacterial genus, reflects easy genetic exchange [80].

Macrolide antibiotics are of little value in *A. baumannii* infections. Azithromycin, but no other macrolide, appears to inhibit mucin production, suggesting efficacy against ventilator-associated pneumonia. This antibiotic has been shown to have immunomodulatory effects at different points in the inflammatory cascade, modulating cellular functions and signaling processes, so it can be speculated that this may be related to its success in the treatment of ventilator-associated pneumonia in *A baumannii*. According to the MicroBIGG-E database, macrolide resistance in *A. baumannii* is attributed to (i) three 23S rRNA (adenine(2058)-N(6))-methyltransferases, encoded by *erm(B)*, *erm(C)*, and *erm(F)*); (ii) the ABC-F-type ribosomal protection protein Msr(E) or msr(E); and (iii) two macrolide 2′-phosphotransferases encoded by *mph(A)* and *mph(ME)*. The first two classes lead to resistance through target site modification, whereas the third class results in the inactivation of macrolides [59].

### 4.6. Tetracycline Resistance

An effective treatment against infections caused by *A. baumannii* is using tetracyclines (an antibiotic that binds to the 30S subunit of the ribosome inhibiting protein synthesis by interrupting the initiation of translation), where minocycline and doxycycline are included. It has been reported that the use of tetracyclines in combination with other antibiotics has been successful in the treatment of respiratory infections in 71.9% and in blood infections in 87.5%. Nevertheless, as in other bacteria, an efflux system has been described to reduce the accumulation of antibiotics, and it is known as a potent mechanism of drug resistance. In the case of tetracyclines, it has been reported that the frequency of resistance is more than 50%; even studies have determined up to 91.6% resistance to this antibiotic [59,60,81,82].

Three main mechanisms involved in tetracycline resistance have been described: ATP-dependent efflux pumps, tetracycline inactivation by enzymes, and ribosomal protection proteins (RPPs). Efflux pumps belong to the RND (resistance-nodulation-cell division) family, having the characteristic that they are non-specific constitutive pumps and the *adeA*, *adeB*, and *adeC* genes, which encode for a periplasmic adaptor subunit; a permease and outer membrane pump elements, respectively, originate them. The other efflux pumps involved in tetracycline resistance belong to the tetracycline major facilitator superfamily (MFS), in which TetA and TetB are found. The RPPs proteins (TetM, TetW, TetO, and TetS) eliminate the inhibitory effect of tetracycline on protein synthesis due to non-covalent modifications of the ribosomes. In other studies, it has been determined that variants of the *tet(X)* gene rapidly confer resistance to tigecycline, since the monooxygenases Tet (X3), Tet (X4), and Tet (X5) can inactivate this antibiotic, as well as eravacycline, omadacycline, and all theracyclines [59].

The *tetA* and *tetB* genes have been searched for and are found in at least 14–46% and 50%, respectively, of tetracycline-resistant *A. baumannii* strains. The *tetA* gene coding for a resistance protein may be contained in a transposon that has been partially characterized, as well as the *tetR* gene coding for a regulatory protein, while the *tetB* gene is carried by a 5 to 9 kb plasmid conferring multidrug resistance of *A. baumannii* strains. TetM, as previously described, has been associated with tetracycline and minocycline resistance through ribosomal protection [19,59,81].

TetA can act synergistically with efflux pumps belonging to another superfamily called RND. Three systems, AdeABC, AdeFGH:RND, and AdeIJK, function as mechanisms of resistance to tigecycline; the expression of the latter two systems is controlled by AdeL, a LysR-type transcriptional regulator, and AdeN, which is a TetR-type transcriptional regulator [19,81,82,83,84].

The AdeABC system is mainly responsible for resistance in *A. baumannii* strains, which is under the control of another two-component system called AdeRS; point mutations in the *adeRS* operon can increase the expression of the pump and consequently lead to antibiotic resistance. The insertion of a sequence known as ISAba1 in the *adeS* gene also leads to the overexpression of *AdeABC*. Conversely, it has been determined that a deletion in *AdeR*, the other transcriptional factor that regulates the expression of *AdeABC*, reduces the MIC of tigecycline. Transcription of the *adeA* gene is also regulated by the BaeSR system and cell density, affecting susceptibility to tigecycline. AdeABC is also associated with aminoglycoside resistance. Several studies have detected the presence of *adeA* and *adeS* genes in a frequency of more than 60%, and that the percentage may vary due to the pattern of antibiotics used, the type and number of clinical samples analyzed, methodology used, and environmental factors, among others [19,59,60,83].

Deletion mutations in the *trm* gene, which encodes for an S-adenosyl-L-methionine-dependent methyltransferase, and mutations in the reading frame of the *plsC* gene, which encodes for 1-acyl-sn-glycerol-3-phosphate acyltransferase, are associated with decreased susceptibility to tigecycline. In contrast, the sequencing of complete genomes of tigecycline-resistant strains with a deletion in the *adeR* gene has revealed that a mutation in the *trm* gene makes *adeR* mutants resistant to tigecycline. Additionally, the *abpr* gene, which codes for a C13 peptidase, is associated with decreased susceptibility to tetracycline, as a deletion or elimination of this gene increases the permeability of the cell membrane, slowing cell growth and conferring reduced susceptibility to these antibiotics [19].

### 4.7. Oxazolidinone Resistance

Within this class of antibiotics, linezolid was one of the first available for the treatment of infections caused by *A. baumannii* and other bacteria. Oxazolidinones bind to the 50S ribosomal subunit in competition with chloramphenicol and lincomycin, but do not inhibit peptidyl transferase as the other two antibiotics do. It has been reported that they do not inhibit the formation of fMet-tRNA and the enlogation or termination stage; although, in other studies, it has been shown to inhibit the binding of fMet-tRNA to the P site. It should be noted that the main action of oxazolidinones is by binding the P site, inhibiting the initiation complex and the translocation of peptidyl-tRNA from the A site to the P site. In the case of linezolid, the most prevalent mechanisms of resistance described are in addition to the presence of multidrug efflux pumps, the modification of the target site where mutations by base substitutions in the V domain of the 23S rRNA and/or the presence of a transmissible Cfr(B) rRNA 23S methyltransferase can occur [59,84].

According to the CARD (Comprehensive Antibiotic Resistance Database), mutations in the P site of the 50S ribosomal subunit, where peptidyl-tRNA attacks to the developed polypeptide chain occur, generate resistance to linezolid. In addition to the presence of efflux pumps (LmrS, capable of expelling various antibiotics in addition to linezolid), a 23S rRNA methyltransferase-like cfr, known as ClcD, and the *poxtA* gene encoding for a ribosomal protection protein (ABC-F ATP-binding cassete ribosomal protection protein), contribute to oxazolidinone resistance [59].

### 4.8. Aminoglycoside Resistance

Aminoglycosides are drugs of choice for the treatment of infections caused by strains with high resistance profiles. Among the most representative ones are streptomycin, apramycin, tobramycin, gentamicin, amikacin, and neomycin B. They bind specifically at the A site of the 16S rRNA of the 30S ribosome to inhibit protein synthesis and have been used in combination with extended-spectrum β-lactams for the treatment of Gram-negative microorganisms; although, in the treatment of infections caused by *A. baumannii*, it has been determined that high levels of resistance to aminoglycosides can cause serious problems if these are combined [84].

Three mechanisms of aminoglycoside resistance have been described in *A. baumannii*: aminoglycoside-modifying enzymes (AMEs) that weaken the binding capacity of these antibiotics, target site randomization by 16S rRNA methyltransferases, and limited uptake of aminoglycosides following loss of permeability or hyperactivity of efflux pumps [59].

In *A. baumannii*, resistance to aminoglycosides is conferred mainly by the mechanism of modifying enzymes, which can be classified into acetyl, nucleotidyl, and phosphotransferases. Acetyltransferases are known to be enzymes that acetylate amino groups found at various positions in the structure of aminoglycosides in an acetyl-CoA-dependent reaction. Phosphotransferases act as kinases, since they catalyze the ATP-dependent phosphorylation of hydroxyl groups found on aminoglycosides, and modifications made by these enzymes decrease the binding affinity to the target site by reducing the hydrogen-bonding potential of the hydroxyl groups of aminoglycosides with important rRNA residues. Nucleotidyltransferases are enzymes that act by adding AMP from ATP to a hydroxyl group of the aminoglycoside at the 2″, 3″, 4″, 6″, and 9″ positions. All modifications in aminoglycosides lead to the reduction in or elimination of the binding of the molecule to the ribosome. The genes encoding these enzymes are phosphotransferases APH (3′)-VIa (*aphA6*), acetyltransferases AAC (3)-Ia (*aac1*), nucleotidyltransferases ANT (2″)-Ia (*aadB*), and ANT (3″)-Ia (*aadA1*). The genes encoding for aminoglycoside-modifying enzymes are localized and can be transported in class I integrons, transposons, genetic cassettes, and plasmids described in MDR strains. According to different studies, the most prevalent genes are *aadA1*, *aadB*, *aphA6*, and *aacC1*, while, in others, it has been reported that the most frequent are *aac(6′)-Ib*, *aac(3)-I*, *aph(3′)-I*, and *armA*; the least frequent are *aac(6′)-Id* and *rmtA*. It has been suggested that the mobility of these aminoglycoside-modifying enzymes is linked to their origins and may be carried through horizontal gene transfer from actinomycetes that are responsible for the natural production of aminoglycosides [19,59,85,86,87,88,89].

The genes encoding for phosphotransferases *aphA1* and *aph(3′)-IIb* have been correlated with high resistance to amikacin, gentamicin, and tobramycin. Other reports have determined that *aph(3′)-VIa*, *aph(3′)-VIb*, and *aph(3′)-VI* genes are significantly more prevalent among amikacin- and kanamycin-resistant isolates, while *aac(6′)-Ian*, *aac(6′)-Ib*, *aac(6′)-Ib3*, *aac (6′)-I*, and *aac(6′)-Il* genes exhibit resistance against amikacin, kanamycin, and tobramycin [59,86,89].

It has also been reported that 16S rRNA methylases or methyltransferases (*ArmA*, *RmtA, RmtB, RmtC, RmtD, RmtE*, and *NpmA*) in *Acinetobacter* spp. strains confer a high level of resistance to most aminoglycosides except streptomycin, modifying the target site leading to aminoglycoside resistance; in other words, they modify specific rRNA nucleotide residues thereby blocking the binding of the aminoglycoside to its target [19,85,86,89].

**Figure 2:**
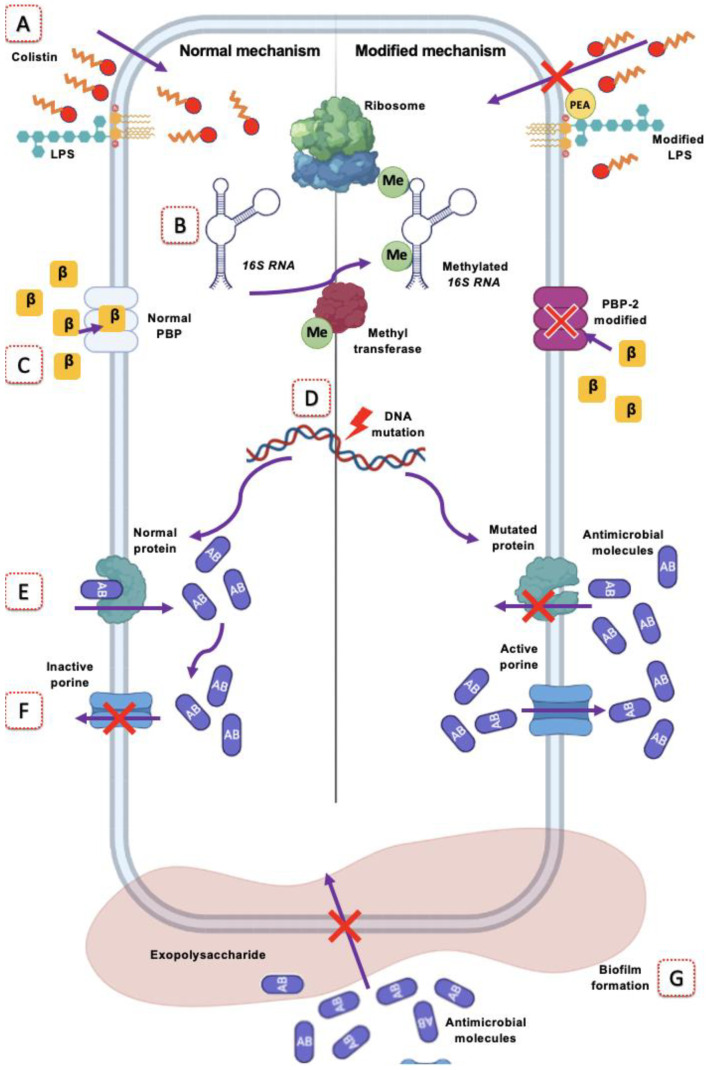
Mechanisms of antimicrobial resistance mediated by target site modification. In *A. baumannii*, the mechanisms it can use to be resistant to antimicrobials are: (**A**) Addition of phosphoethanolamine (PEA) to the lipopolysaccharide (LPS) molecule that confers resistance to colistin; (**B**) Methylation (Me) of 16S RNA mediated by methyltransferase, which prevents action with the antimicrobial; (**C**) The modification of penicillin-binding protein (PBP) to PBP-2A, which has a low affinity for β-lactams (β); (**D**) Spontaneous mutations in DNA causing antimicrobial receptor or antimicrobial recognition (AB) proteins to be modified and fail to perform their function; (**E**) The protein to which the antimicrobial binds is modified; therefore, the antimicrobial cannot interact with the protein. (**F**) The antimicrobial inside the bacterium accumulates causing the death of the bacterium by an excess; through the use of efflux pumps, the bacterium can expel the antimicrobial. (**G**) The production of exopolysaccharide and the subsequent formation of biofilm prevents the contact of some families of antimicrobials with the bacterial structure, which generates resistance. Created at BioRender.com accessed on 3 June 2022.

Figure 2 summarizes the different changes in the target sites that *A. baumannii* makes to evade the action of various antimicrobials.

## 5. Treatment Options for *A. baumannii* Infections

### 5.1. Current Treatment Options

*A. baumannii* with extensively drug resistance (XDR) or pan-resistance (PDR) is usually resistant to colistin and carbapenems, so treatment options are limited. The use of comprehensive strategies has been suggested for the eradication of infections by this microorganism; there is no main line in therapeutics, so many factors must be taken into consideration [86].

As a comprehensive strategy, the following is recommended [59,90]:

- Use antimicrobial associations.
- Administer them by optimizing the pharmacokinetic/pharmacodynamic (PK/PD) ratio.
- Give preference to antimicrobials that retain some degree of in vitro activity.
- Optimize other therapeutic measures, e.g., surgical debridement or removal of infected tissues or devices.
- Be sure that it is an infection instead of a colonization before starting the treatment.

The site of infection should be considered in the choice of antimicrobials to be used in monotherapy (only if the microorganism is fully sensitive) or in association with others. In general, the following is recommended:

■ Bacteremia: In cases associated with catheters or intravascular devices, give priority to their removal. Start the scheme with carbapenems, aminoglycosides, and rifampicin.
■ Pneumonia: Give preference to combined therapy for community-acquired pneumonia; for ventilator-associated pneumonia, when the patient has endotracheal intubation and mechanical ventilatory support, the patient should be placed in a semi-sitting position between 30° and 45°, preferably in a kinetic bed, which provides position changes with head elevation, in order to reduce the production of secretions. There is not enough evidence to support the generalized use of endotracheal cannulas impregnated with antiseptics for the reduction of VAP. (GPC IMSS624-13).
■ Urinary Tract Infection: Give preference in the plan to aminoglycosides and carbapenems. Use the Foley catheters for as long as necessary and remove them as soon as possible.
■ Central Nervous System Infection: Give preference in the plan to carbapenems and rifampicin for systemic use. Evaluate intraventricular or intrathecal use with aminoglycosides; however, their use is controversial since there are not much data in this regard.
■ Abdominal infection. In this site, it is essential to give priority to surgical treatment; start the plan with tigecycline, sulbactam, carbapenems, or aminoglycosides.
■ Skin and soft tissue infection: Prioritize surgical treatment by removing dead and contaminated tissue; initiate scheme with tigecycline, carbapenems, and aminoglycosides.
■ Infection or Osteoarticular: Give priority to surgical treatment by scraping bone and dead tissue; perform surgical lavage. Give preference in the plan to aminoglycosides and carbapenems.

For the consideration of antimicrobial activity, the MIC to the different antimicrobials should be observed, even when the susceptibility study reports the presence of intermediate susceptibility or resistance.

The following antimicrobials are considered active (CPG IMSS624-13):

○ Sulbactam, if MIC is less than or equal to 32 mg/L;
○ Meropenem or imipenem, if MIC is less than or equal to 16 mg/L;
○ Colistin: Less than or equal to 1 mg/L;
○ Tigecycline, if MIC is less than or equal to 4 mg/L.

It is important to take into consideration that none of the new β-lactam–β-lactamase-inhibitor antibiotic combinations are active against *A. baumannii*, if it is resistant to carbapenems [87]. In addition, an aminoglycoside, semi-synthetic derivative of sisomycin, has no better activity compared to the other alternative antibiotics of the same family; nevertheless, when associated with carbapenems, it has a very promising synergistic effect [19]. Likewise, *A. baumannii* is intrinsically resistant to fosfomycin [59,91].

New options currently available for resistant *A. baumannii* include minocycline, eravacycline, and cefiderocol. Eravacycline is more potent compared to tigecycline and may be an option against some tigecycline-resistant strains of *A. baumannii*. Minocycline has also been proposed as an option and has been used against carbapenem-resistant isolates, but its role and activity against resistant strains is unclear, especially considering its undefined susceptibility cut-off points and lack of current pharmacokinetic and pharmacodynamic studies [59,92].

Cefiderocol, a cephalosporin with bactericidal effect, acts by inhibiting the cell wall synthesis of Gram-negative bacteria by binding to penicillin-binding proteins; however, it is unique because it enters the bacterial periplasmic space because of its siderophore-like property and has a higher stability against β-lactamases. The chemical structure of cefiderocol is similar to both ceftazidime and cefepime, which are third and fourth generation cephalosporins, respectively, but with high stability to a variety of β-lactamases, including AmpC and extended-spectrum β-lactamases (BLEEs). Cefiderocol is active against most *A. baumannii*, but resistant strains have already been reported and its availability is limited in many countries [59,92].

Synergistic combinations based on polymyxins, for example, with rifampicin, carbapenems, ampicillin/sulbactam, fosfomycin, glycopeptides, tigecycline, and minocycline, are the most studied, but have been tested predominantly against polymyxin-sensitive but carbapenem-resistant *A. baumannii*, and in most studies, no clinical benefits have been found yet.

Polymyxin B has pharmacokinetic advantages over colistin and is less nephrotoxic, except in lower urinary tract infections. The combination of colistin with rifampicin has been successfully used against colistin-resistant *A. baumannii* pneumonia and colistin-resistant *A. baumannii* postsurgical meningitis [93].

The synergy is remarkable between colistin and agents that are not active against Gram-negative bacteria, such as glycopeptides, including vancomycin, teicoplanin, and telavancin, which exert their activity by inhibiting peptidoglycan synthesis, but do not penetrate the membrane of Gram-negative bacteria and are considered inactive against this class of pathogens. However, the disruption of the outer membrane may allow them to reach their targets in these bacteria [93].

### 5.2. Alternative Therapies without Antibiotics

Phage therapy is encouraging; in many cases, good clinical results can be observed, while its safety seems to be unquestionable. To date, randomized clinical trials have recently confirmed the safety of the therapy, even at very low phage titers, by reducing the bacterial load in treated patients. Clearly, more clinical trials are urgently needed to confirm the value of phage therapy against this pathogen according to evidence-based medicine [90,91]. Attention has been focused on bacteriophage-encoded endolysin. Endolysin is a lytic enzyme that degrades the cell wall of bacterial hosts and shows promise as a new class of antibacterial with a unique mode of action; an example is endolysin from *A. baumannii* bacteriophage ØABP-01 that degrades the crude cell wall of strains and elevates antibacterial activity when combined with colistin [19,94,95].

There are other molecules of peptide nature with an alpha helix structure where in silico and in vitro studies have shown antimicrobial activity by attacking the membrane or intracellular structures of multi-resistant *A. baumannii*; among them we have Melittin, Histatin-8, Omega76, AM-CATH36, Hymenochirin, and Mastoparan [96].

A summary of possible therapies against infections caused by *A. baumannii* is shown in Table 2.

Some other known molecules have been evaluated for in their vitro antimicrobial effects, for example, the SecA inhibitor (Rose Bengal), which inhibits the periplasmic translocation of these class D β-lactamases that hydrolyze carbapenems, whereby imipenem or meropenem combined with Rose Bengal shows synergistic effects. Likewise, the use of gallium nitrate, gallium protoporphyrin IX, as well as some D-amino acids, such as D-His and D-Cys, which inhibit bacterial growth, biofilm formation, and adherence to eukaryotic cells in *A. baumannii* have been investigated [19]. Another possible treatment option against *A. baumannii* infections is bacteriocins.

Bacteriocins are a group of peptides produced by both Gram-negative and Gram-positive bacteria that have antimicrobial activity against bacteria related to the bacteriocin producer. The production of bacteriocins allows the producer to survive in a highly competitive polymicrobial environment, and it is known that these molecules exhibit a large antimicrobial spectrum depending on the peptide that could target several bacteria [97,98]. Many works focus attention on the use of bacteriocins as an antimicrobial agent against infections caused by high-antimicrobial-resistance bacteria, such as *E. coli*, *P. aeruginosa*, and *K. pnuemoniae*, to name a few [97]. In the case of *A. baumannii*, there is not as much information on this use, but Leblanc et al., in 2014, reported the potential activity of ST4A, a bacteriocin produced by *Enterococcus mundtii*; this peptide also has activity against *P. aeruginosa*, *E. faecium, E. faecalis, S. aureus*, and *S. pneumoniae*. [99].

Nowadays, there is a clinical trial for the potential use of the bacteriocin nisin made by Intrabiotics (Mountain View, CA, USA) to assess its inhibitory effects on pathogens associated with VAP where *A. baumannii* is included [100].

## 6. Conclusions

Infections caused by *A. baumannii* are of relevance in the healthcare setting, due to the high frequency of isolates of multi-resistant strains. These infections can lead to the death of patients when they are not well treated. Part of this antimicrobial resistance is caused by different mechanisms used by this bacterium to evade the action of antimicrobials that are used as treatment against it. Among these mechanisms is the modification of the target sites in which the action of antimicrobials is directed, which together causes resistance to a wide range of antimicrobials. This leads to a decrease in therapeutic options to fight these infections, so it is necessary to know in detail these mechanisms to contribute to the development of new therapeutic options against these infections.

## Figures and Tables

**Table 1:** Antimicrobial resistance described in *A. baumannii*.

Antimicrobial Family		Antimicrobials
β-lactams	Penicillins	Ampicillin ^a^
		Amoxicillin-clavulanate ^a^
		Ticarcillin Mezlocillin Piperacillin
		Piperacillin-tazobactam
	Cephalosporins	Cefoxitin Cefotetan Cefepime Ceftazidime Cephalothin Ceftriaxone Cefotaxime
	Monobactams	Aztreonam ^a^
	Carbapenems	Ertapenem ^a^ Imipenem Meropenem
Amphenicols		Chloramphenicol ^a^
Phosphonates		Fosfomycin ^a^
Sulfonamides and diaminopyrimidines		Trimetroprim ^a^ Trimethoprim/sulfamethoxasol
Aminoglycosides		Amikacin Gentamicin Trobamycin
Macrolides		Erythromycin Azithromycin
Tetracyclines		Glycylcyclines Tigecycline Doxycycline Minocycline
Fluoroquinolones		Ciprofloxacin Norfloxacin Levofloxacin Moxifloxacin Gatifloxacin
Nitrofurans		Nitrofurantoin
Polymyxins		Polymyxin B Colistin

^a^ Intrinsic resistance in *A. baumannii* [36]. Modified from the literature [23,26,33,37,38,39,40,41,42,43,44].

**Table 2:** Possible therapies against *A. baumannii*.

Criteria	Condition	Options			
Using Tigecycline (4) as a backbone	If MIC is less than or equal to 2 mg/L (sensitive)	Associated with aminoglycosides (gentamicin or amikacin)	Associated with sulbactam	Associated with rifampicin	Associated with carbapenem
	If MIC is equal to 4 mg/L (intermediate)	Associated with sulbactam + carbapenem	Sulbactam + fosfomycin	Sulbactam + rifampicin	Sulbactam + aminoglycoside (amikacin/gentamicin)
	If MIC is greater than 8 mg/L (resistant), do not use tigecycline. Substitute MINOCYCLINE	Carbapenem (imipenem or meropenem) + sulbactam + rifampin	Carbapenem + sulbactam + aminoglycosides	Carbapenem + aminoglycoside rifampicin	
Using β-lactam-β-lactamase inhibitors	Do not use if the strain is carbapenem-resistant.	Meropenem/vaborbactam	Imipenem/relebactam	Ceftazidime/avibactam	Ceftolozane/tazobactam or aztreonam/avibactam
For carbapenem-resistant strains	*A. baumannii* that does not produce MBL	Ampicillin/sulbactam and trimethoprim/sulfamethoxazole	Ampicillin-sulbactam + polymyxins (polymyxin b/colistin)	Sulbactam/avibactam	Ampicillin/sulbactam with ceftazidime/avibactam
New alternatives					
New antimicrobials	Minocycline alone or in association	Eravacycline	Cefiderocol (resistant strains have been found and availability is limited		
Based on phages and probiotics	vB_Ab-M-G7	Bϕ-C62	Βϕ-R2096	Endolysins of phage ØABP-01	Bifidobacterium brief on digestive tract infections
Molecule-based	DS-8587 is a new fluoroquinolone that acts by inhibiting DNA topoisomerase	BAL 30072 monosulfactam; active against many Gram-negative bacteria, including those producing metallo-β-lactamases and KPC, and has a synergistic effect with carbapenems	GC-072 in preclinical phase. It is an oxoquinolizine compound	Gallium nitrate or gallium protoporphyrin IX whose activity is to sequester Fe ions	Rose Bengal (SecA inhibitor) in combination with imipenem or meropenem
Bacteriocins	ST4A produced by E. mundtii	Nisin in clinical trials on pathogens associated with VAP			

Taken and modified from [19,92,93,94,97,98,99,100,101].
